# 83-year-old Woman with a Fever and Emesis

**DOI:** 10.5811/cpcem.2018.10.40507

**Published:** 2018-10-16

**Authors:** Shelly Birch, Sarah B. Dubbs, Laura J. Bontempo, Zachary D.W. Dezman

**Affiliations:** * University of Maryland Medical Center, Department of Emergency Medicine, Baltimore, Maryland; † University of Maryland School of Medicine, Department of Emergency Medicine, Baltimore, Maryland

## CASE PRESENTATION (DR. BIRCH)

An 83-year-old Filipino woman was brought to the emergency department (ED) by family for chief complaints of fever and emesis. Much of the patient’s history and review of systems was obtained through her daughters, as the patient only spoke Tagalog and had severe dementia. The patient lives with her daughters who are her primary caregivers. The patient’s husband is deceased. The daughters report that the patient was at her baseline mental status the night before. This morning they found her warm to the touch and obtained an oral temperature of 38.3° Celsius. She was constantly shifting her gaze about the room and appeared mildly distressed. She then had one episode of non-bloody, non-bilious emesis, so the daughters decided to bring her to the ED for evaluation.

The patient primarily communicates through hand gestures, incomprehensible sounds, or nonsensical words. She has not missed any doses of her medications, nor has she had any cough, urinary frequency or incontinence, any changes in her bowel habits, focal weakness, or rashes. Her daughters report no evidence of abdominal pain, dysuria, back or flank pain, chest pain, or lightheadedness.

The patient has a past medical history of hypertension, diabetes mellitus, myocardial infarction with stent placement, and hyperthyroidism. The patient is a retired teacher but has not worked in years. She has not had any surgeries. She does not drink alcohol, smoke cigarettes, or use illicit drugs. Her medication list includes glimepiride, metformin, gabapentin, pioglitazone, hydrochlorothiazide, lisinopril, aspirin, atorvastatin, ticagrelor, potassium supplements, methimazole, risperidone, trazadone, donepezil, escitalopram, and memantine. Her medicines are administered by her daughters and stored in a cabinet by her bed along with some salves, creams, and medicinal oils. The family reports that the patient has no known drug allergies.

Physical examination revealed a thin, frail, elderly Asian woman lying calmly on the stretcher. She had a temperature of 36.8° Celsius, a blood pressure of 185/85 millimeters of mercury, a pulse of 112 beats per minute, a respiratory rate of 36 breaths per minutes and an oxygenation saturation of 98% on room air. Her body mass index was 21. Her head was atraumatic and normocephalic. Her extra-ocular movements were intact and her pupils were four millimeters in diameter, round, equal, and reactive to light. Her oral mucosal membranes were moist. She was tachycardic on exam, but there were no audible murmurs, rubs or gallops. She had normal breath sounds bilaterally. She was clearly tachypneic and had mild subcostal retractions. Her abdomen was soft and normal bowels sounds were heard. She was not distended or tender, and she did not have any rebound, guarding or organomegaly. All four extremities were warm, well perfused and without any tenderness to palpation. Neurologically, she was not oriented to self, place, or time, and would only follow simple commands.

**Image 1:**
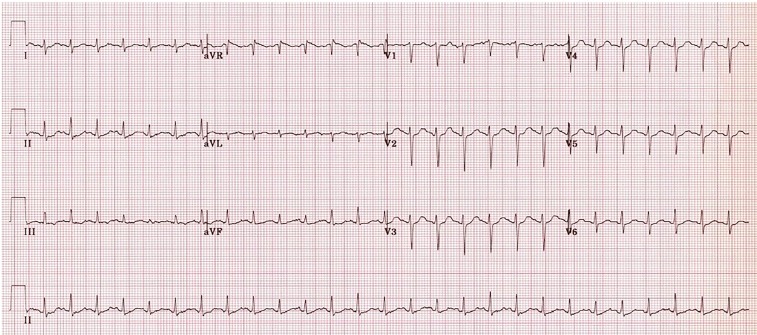
Electrocardiogram of an 83-year-old woman with fever and emesis.

**Image 2:**
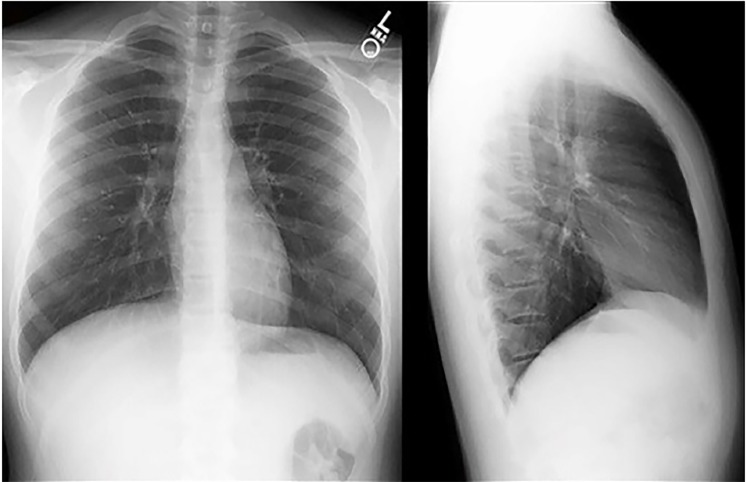
Chest radiograph of 83-year-old woman with fever and emesis.

Laboratory results are shown in Table 1. Her electrocardiogram is shown in Image 1, and a chest radiograph (CXR) in Image 2.

## CASE DISCUSSION (DR. DUBBS)

Geriatric patients with dementia are among the most challenging patients to treat in the ED. They present often with diffuse or non-specific complaints with broad differentials (such as fever or altered mental status). It can be easy to order a litany of labs and imaging, hoping that something comes up positive. Casting a wide net in this situation is not the pitfall; rather, it is encouraged. But the real challenge lies in maintaining a sense of diagnostic vigilance.

What is diagnostic vigilance? Diagnostic vigilance is not settling for a diagnosis of urinary tract infection in an altered 74-year-old woman with 5–10 white blood cells and 25–50 squamous cells per high-power field. Diagnostic vigilance is not settling for a diagnosis of dehydration in an 80-year-old man with acute kidney injury. The word *vigilant* is defined as “alertly watchful especially to avoid danger.”^1^ When we are presented with a patient who cannot provide much history, as in the case presented here, we must be alertly watchful, searching for clues in the history, the physical examination, tests, and re-evaluations to uncover the true diagnosis. So, let’s approach this case of an 83-year-old woman presenting with fever and vomiting with some healthy diagnostic vigilance.

Dr. Birch paints a picture of a thin, frail, elderly Asian woman who is notably hypertensive, tachycardic, and tachypneic with a respiratory rate in the mid-thirties. I am pleasantly surprised, albeit slightly perplexed, that her oxygen saturation is 98% on room air and that her lungs are clear to auscultation (more on that later). Likewise, her soft and non-tender, non-peritoneal abdomen is reassuring in the light of her episode of vomiting prior to arrival. Very quickly, several life-threatening diagnoses such as heart failure with pulmonary edema, perforated viscous, and acute mesenteric ischemia fall away from the top of the differential list.

The patient’s past medical history provides the all-important background on which to visualize our patient. This frail, elderly, retired teacher is on multiple medications for hypertension and coronary artery disease. She is treated with oral medications for diabetes and presumably diabetic neuropathy, takes methimazole for hyperthyroidism, and is on multiple anti-depressants, antipsychotics, and centrally active, Alzheimer’s disease medications. On top of this polypharmacy, the patient appears to have access to medicinal oils and salves (medicinal ointment). Could her hyperthermia, hypertension, tachycardia, and altered mentation be due to thyroid storm? Could there be some kind of medication interaction or toxicity at play?

The patient seems to have a very attentive family that cares for her at home. They are clearly concerned about the fever and episode of vomiting that prompted the ED visit. Mention is also made of a change in her baseline mental status – appearing distressed compared to her usual self – which now adds the cognitive framework of altered mental status to the table.

The differential diagnosis for altered mental status is extensive. Initial assessment of patients with altered mental status should look for vital signs indicative of obvious causes such as hypoxia, hypercapnea, hypoperfusion from shock states, hypo- or hyperglycemia, and environmental hypo- or hyperthermia. When moving on to further investigation, it is helpful to categorize the possibilities into a few broad categories: primary neurologic, toxic/metabolic, infectious, and other causes (Table 2). Etiologies to be considered in the primary neurologic category are intracranial hemorrhage, stroke, and seizure. Toxic/metabolic considerations include alcohols and recreational drugs, medication toxicities, carbon monoxide, hyperglycemia, hypo- or hyperthyroidism, electrolyte abnormalities of sodium, potassium, calcium, or others, acidemia, and uremia. Any infection can cause altered mental status as well, especially in the elderly. It is important to consider meningitis, encephalitis, pneumonia, urinary tract infection, bacteremia and sepsis. Finally, psychiatric disorders should be diagnoses of exclusion after organic etiologies are ruled out.

Now, let’s take this broad, altered mental status differential diagnosis and apply the particulars of this patient. What are the pertinent positive and negative findings on her physical examination and work-up? For the initial assessment, we know that she is not hypoxic. She is confused but alert and has a high respiratory rate, so hypercapnea is not likely. Her level of alertness also makes hypoglycemia unlikely, but a point-of-care glucose would be helpful. Finally, her temperature is not indicative of hypo- or hyperthermia.

Do any of her findings point to a primary neurologic cause? She does not have focal neurologic deficits that would indicate an ischemic or hemorrhagic stroke. She is awake and able to follow simple commands, so seizure is not the diagnosis either.

Do any of her findings point to a primary toxic or metabolic cause? Alcohols could certainly be at play. She does have a small anion gap, which could be caused by a toxic alcohol such as methanol, ethylene glycol, or propylene glycol. Isopropanol presents with profound inebriation/coma, cerebellar signs, and hemorrhagic gastritis, which our patient does not have. Recreational drugs are not impossible, but are less likely given this patient’s social situation; plus she does not have pupillary or other exam findings consistent with classic, drug-ingestion toxidromes. Carbon monoxide is also less likely, as it seems that family members, who reside with her, are asymptomatic. When it comes to medications, the patient has many that could be responsible for her altered mental status. Notably, her tachycardia, hypertension, vomiting, and altered mental status could be caused by escitalopram toxicity. Similar symptoms could be seen as well with thyrotoxicity if she stopped taking her methimazole. Aspirin toxicity may also present with the same tachycardia and tachypnea. Finally, labs effectively rule out major electrolyte abnormalities and uremia. In reviewing the venous blood gas for acidemia as a metabolic cause of the mental status, I find it to be surprisingly normal in light of the patient’s slight anion gap metabolic acidosis and mildly elevated lactate of 2.1. Could there be a secondary process going on there?

Do any of her findings point to a primary infectious cause? With reported fever and the abnormal vital signs in this elderly patient, a sepsis workup is absolutely indicated. I would examine her for meningismus and strongly consider a lumbar puncture to look for central nervous system (CNS) infection if no more-convincing diagnoses are found. Her urine does not appear infected, and there are no signs of pneumonia on her chest radiograph. Could the vomiting be a sign of abdominal infection? Her abdominal exam is reassuring, but that is never guaranteed in the elderly population.

Do any of her findings point to a primary psychiatric cause? Again, psychiatric causes are a diagnosis of exclusion.

Several possible diagnoses came to the surface by going through the altered mental status differential. To review, these are toxic alcohols, escitalopram (selective serotonin reuptake inhibitor) toxicity, thyrotoxicosis, aspirin toxicity, intra-abdominal infection, and CNS infection.

I introduced the concept of diagnostic vigilance at the beginning of this section. Through the process of considering this patient’s presentation within the framework of an altered mental status differential diagnosis, I strived to maintain that vigilance, remaining alertly watchful as would an investigator searching for clues at a crime scene or finding inconsistences in the stories of suspects. A couple aspects of the case were noticeably unexpected in this patient’s presentation. I was surprised that she was not hypoxic and had clear lungs and a negative CXR despite being very tachypneic. I was surprised that her venous blood gas was within normal range when I expected to see acidemia from the anion gap and lactate. Could these two things that both seem like inconsistencies be made consistent, if related? Yes.

The patient has a primary anion gap metabolic acidosis, which is not reflected in the venous blood gas because she also has a primary respiratory alkalosis. One single diagnosis explains these findings, as well as her tachycardia, fever, and altered mental status. That diagnosis is aspirin overdose, also known as salicylate toxicity. The patient is on aspirin for her cardiovascular disease and has potential access to additional salicylate in the creams, salves, and oils stored at her bedside. Therefore, the diagnostic study that I would perform is a serum salicylate level to confirm the diagnosis of salicylate toxicity.

## CASE OUTCOME (DR. BIRCH)

**Image 3:**
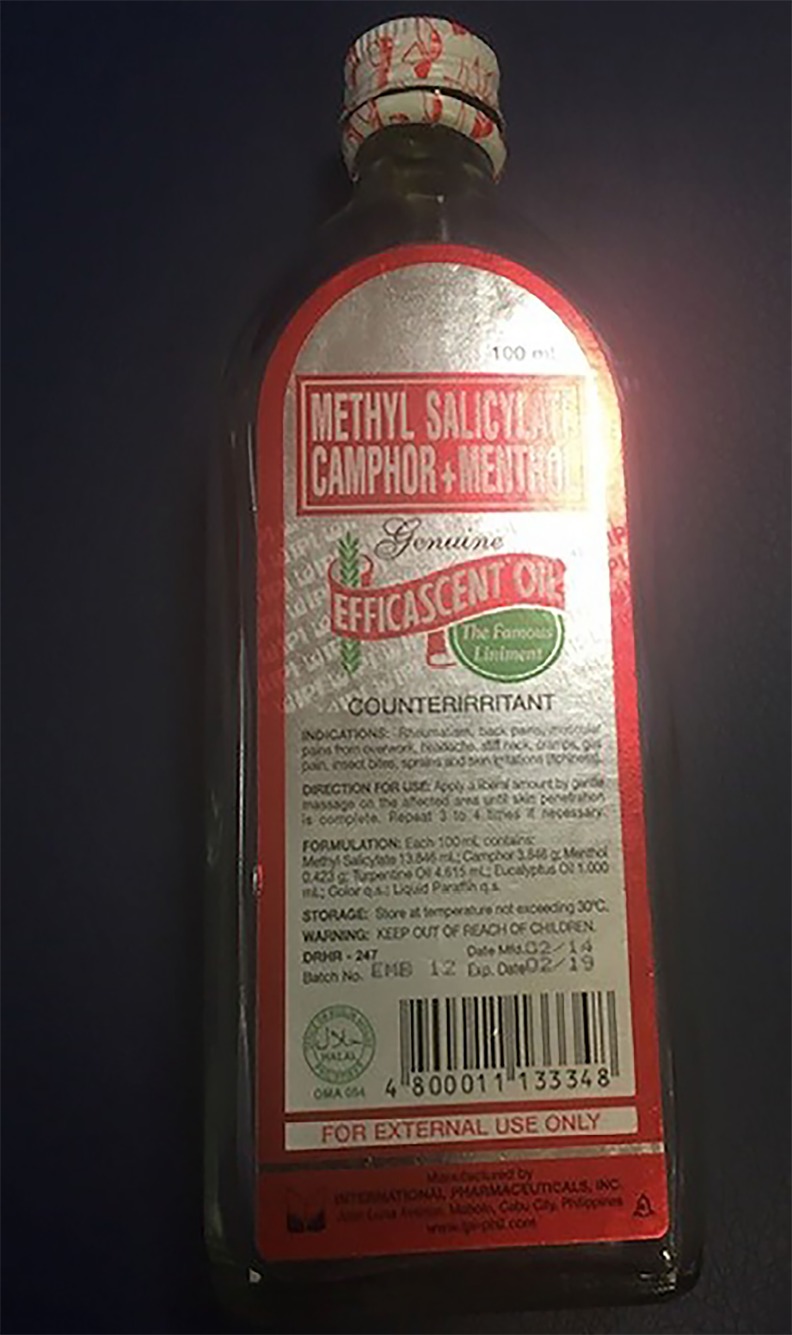
Empty container of Efficascent Oil, a topical pain-reliever containing camphor and methyl salicylate.

The patient was found to have a salicylate level of 45 milligrams per deciliter (mg/dL). Shortly after the level returned, another family member arrived with an empty bottle from the patient’s medicine cabinet that had been full the day before (Image 3). Our patient had ingested approximately three-quarters of a 100-milliliter (ml) bottle of methyl salicylate camphor and methyl salicylate oil. This was calculated to be more than 18 grams of salicylate and the patient was diagnosed with acute salicylate poisoning. She was started on a bicarbonate drip and admitted to the hospital, where her peak salicylate level was found to be 53.7 mg/dL.

## CASE DISCUSSION

In the 1960s and 1970s salicylate toxicity was the leading cause of fatal overdoses. Salicylate toxicity has declined since then, especially in children, due to increased public awareness about Reye’s syndrome and child-resistant packaging.^2^ Yet the entity is still very relevant to emergency medicine as over 24,000 salicylate overdoses occurred in 2014.^3^

The term “salicylates” refers to a large group of medications. For example, salicylic acid is a topical medication used to treat acne or to eliminate warts, but it is too irritating to the gastric mucosa to be ingested directly.^2^ Methyl salicylate is a formulation that is almost always used topically to alleviate aches and pain. Salicylate containing compounds include oil of wintergreen (a topical oil solution), Bengay®, salicylic acid (a wart remover) and even Pepto Bismol®, (a common medicine for indigestion) which contains bismuth subsalicylate 262 mg per 15 ml. Luckily, salves are very poorly absorbed cutaneously, so it takes coating oneself in a copious amount of ointment to achieve toxic levels. However, there was a 2002 toxicology case report of a naturopath who used methyl salicylate to treat psoriasis, which caused systemic toxicity.^4^ In this case absorption was enhanced due to the large body surface area used with an occlusive dressing. There are also combined medicines that include aspirin with other oral medicines such as Percodan® and Excedrin®.

Salicylates act through three main mechanisms: inhibition of the cyclo-oxygenase (COX) enzymes, stimulating chemoreceptors in the brain and alteration of cellular metabolism.^5^ The COX-1 and COX-2 enzymes catalyze platelet aggregation and prostaglandin synthesis. Aspirin and related salicylates cause nausea and vomiting by inhibiting these enzymes, resulting in decreased prostaglandins, which protect the gastric mucosa.^6^ COX inhibition also causes platelet dysfunction, which theoretically increases a patient’s risk of bleeding, though this is rarely a clinical presentation of salicylate toxicity.^7^ The nausea and vomiting caused by the decreased prostaglandin synthesis is augmented by stimulation of the chemoreceptor zone in the medulla.^8^ Salicylates also activate the respiratory center, causing a respiratory alkalosis.^9^ Aspirin and other salicylates uncouple oxidative phosphorylation, which causes a metabolic acidosis through an increase in lactic acid and organic acids.^10^

In its mild toxicity – levels of 40–60 mg/dL – there may be tinnitus caused by sensorineural deafness (from salicylates’ neurotoxic effects) and possibly mild tachypnea, nausea and vomiting.^10^,^11^ At moderately toxic levels of 60–80 mg/dL, the toxidrome presents with respiratory distress and CNS dysfunction, which can manifest as lethargy or, more commonly, agitation.^10^ In addition to the directly stimulated tachypnea and hyperpnea, dyspnea may be caused by salicylate-induced pulmonary edema (SIPE). SIPE occurs due to the impaired permeability

In chronic toxicity, patients usually appear more ill, have fewer classic presentations and lower salicylate levels. Patients may appear to be septic, in diabetic ketoacidosis, or rarely in decompensated heart failure.^16^,^17^ For such patients you should have a lower threshold to dialyze.

Understanding absorption and excretion of salicylates is key to understanding the clinical manifestations and treatment of their toxicity. Normally, therapeutic doses of aspirin are immediately absorbed in the stomach and converted to salicylate.^18^ Approximately 90% of salicylate is metabolized through hepatic conjugation, which can be saturated, and only 10% of total elimination is achieved renally. At therapeutic doses, peak concentrations occur in one hour, with a half-life of two to four hours.^2^ In contrast, large doses of salicylates can delay peak levels and delay absorption due to bezoar formation, enteric-coated or extended-release versions, and pylorospasm^19^,^20^ Once hepatic mechanisms of metabolism are saturated, the rate-limiting step for excretion is the much slower renal route; peak levels may not occur for 30 hours.^21^ Because of this, it is very important to repeat any positive level until it is downtrending twice to ensure the clinician does not miss a delayed peak level.

Non-polar molecules diffuse easily over cell membranes. Because salicylate is a weak acid, it can easily take an uncharged (pronated) form, allowing it to cross cellular barriers such as the blood-brain barrier or the renal tubule. When salicylate is charged (deprotonated), it stays in the plasma and is not able to damage organs as easily. The main determinant of whether salicylate is charged is the pH of its environment. Salicylates will become pronated (and thus uncharged) at lower pHs, which is part of the toxicity itself. Once the metabolic acidosis develops, more of the salicylate is in its protonated form and able to create significant toxicity.^22^ This principle is important for understanding both why we order certain labs and how we treat salicylate toxicity.

Important labs to order while evaluating someone with possible salicylate toxicity are a salicylate level and a blood gas, which allow assessment of the toxicity severity and response to therapy. It is also important to measure potassium to monitor for hypokalemia, which would interfere with one of the mainstays of therapy: alkalinization of the urine. Hypokalemia promotes the resorption of potassium in the distal tubule via a potassium-hydrogen ion pump.^22^ As the kidney tries to retain potassium, the urine becomes more acidotic and does not favor urinary excretion of the salicylate. Glucose is important to measure because neuroglycopenia can happen at normal glucose level,^14^,^15^ and it is preferable for a toxic patient to be normo- to hyperglycemic.

Other important studies include creatinine to evaluate for kidney function, coagulation studies to assess liver function, anion gap, and imaging (CXR and head computed tomography) to determine other possible causes of the patient’s presentation.

When treating these patients we must first, of course, address the patient’s airway and breathing. The most important principle to remember regarding intubation in salicylate toxicity is to avoid it if at all possible.^23^ In even the small amount of time the patient is apneic, respiratory acidosis results and more of the salicylate is protonated, allowing it to enter the CNS. When addressing the patient’s circulation, the clinician must realize that many patients with salicylate toxicity will be dehydrated due to vomiting and insensible losses secondary to their tachypnea.^10^ Patients will likely require aggressive fluid resuscitation. This should only be delayed in cases of cerebral or pulmonary edema.

The main principles of treatment after attending to airway, breathing, and circulation are decontamination and alkalization of the blood and urine.^24^ Immediately decontaminating with one gram/kilogram of activated charcoal, orally, up to 50 grams is a critical first step.^25^ Ideally, this should be given within two hours of ingestion; however, it should always be given due to the possibility of bezoar formation, enteric-coated tablets, and pylorospasm.^2^ After the initial dose, activated charcoal can be given 25 grams orally every two hours for three doses or 50 grams orally every four hours for two doses.

As discussed previously, the charged form of salicylates will more readily stay in the plasma and be amenable to excretion, whereas the uncharged form will more easily cross into cells to interfere with metabolism and the brain and cause damage. Because the ion will retain its uncharged form in an alkaline environment, the mainstay of treatment is to “trap” the salicylate ion in the plasma and urine by alkalizing them. Alkalization is achieved with a bolus (1–2 milliequivalents per kilogram [meq/kg]) and infusion (100–150 meq in dextrose 5% sterile water) of sodium bicarbonate with titration to a urine pH of 7.5 to 8.^22^ As previously discussed, potassium levels must be monitored and normokalemia maintained to effectively keep the urine basic. Urine alkalization to a pH of 7.5–8.0 increases urinary excretion of salicylates significantly.^26^ It is important to also monitor blood gases in order to avoid severe alkalemia (arterial pH >7.6). Indications for hemodialysis include volume overload preventing sodium bicarbonate therapy, pulmonary edema, cerebral edema, kidney failure that impairs salicylate elimination, salicylate levels over 80 mg/dL, and severe acidemia.^10^ Consultation with nephrology is essential even when patients do not appear to immediately require hemodialysis, as it will help facilitate treatment in case the patient decompensates.

The importance of this elusive diagnosis is highlighted by the reflections of two physicians:

“Twenty-five years ago, a lady jumped off a bridge, and survived her trauma. She was admitted and appeared to go into sepsis with high fever but later died of an aspirin overdose which had not been checked for. She was trying to end her life for sure and it worked.”^27^

“I had an unfortunate lady a few years ago who presented in status epilepticus. Airway obtained early with Ativan[™] for seizures, blood gas obtained with pH around 7.0. Boyfriend provided history of missing bottle of aspirin as she proceeded to arrest.”^28^

## FINAL DIAGNOSIS

Acute salicylate toxicity.

## KEY TEACHING POINTS

1. Cast a wide diagnostic net including ingestion when confronted with elderly patients, especially those with dementia.
2. When clinically suspicious of salicylate toxicity, do not accept a “therapeutic level;” always repeat until the level is declining for two serial values.
3. Salicylate toxicity causes fever, metabolic acidosis and respiratory alkalosis by uncoupling oxidative phosphorylation and stimulating the respiratory centers in the medulla.
4. Decontamination with charcoal should be initiated rapidly, and intubation should be avoided as long as possible.
5. Keep the patient hyperglycemic, as the CNS may be hypoglycemic, and alkalize the patient’s serum and urine with sodium bicarbonate.
6. Speak to nephrology early in the patient’s care because dialysis can be lifesaving.

Documented patient informed consent and/or Institutional Review Board approval has been obtained and filed for publication of this case report.

## Figures and Tables

**Table 1:** Laboratory values for 83-year-old woman with fever and emesis.

Complete blood cell count
White blood cells 8.7 K/μL
Hemoglobin 11.6 g/dL
Hematocrit 35.2%
Platelets 246 K/μL
Serum Chemistries
Sodium 148 mmol/L
Potassium 4.1 mmol/L
Chloride 106 mmol/L
Bicarbonate 23 mmol/L
Blood urea nitrogen 40 mg/dL
Creatinine 1.41 mg/dL
Glucose 106 mg/dL
Calcium 10.8 mg/dL
Magnesium 1.7 mg/dL
Phosphorous 3.8 mg/dL
Total Protein 8.6 g/dL
Albumin 4.7 g/dL
Aspartate Aminotransferase 30 u/L
Alanine Aminotransferase 25 u/L
Alkaline Phosphatase 96u/L
Total Bilirubin 0.5 mg/dL
Ammonia <9 mg/dL
Coagulation Profile
Prothrombin Time 14.0 seconds
Partial Thromboplastin 28 seconds
International Normalized Ratio 1.0
Urinalysis
Appearance Clear
pH 7.0
Ketones Negative
Bilirubin Negative
Protein Negative
Nitrite Negative
Red Blood Cells 26–50/HPF
White Blood Cells 0–2/HPF
Venous Blood Gas
pH 7.36
pCO2 30 mmHg
pO2 35 mmHg
HCO3 29 mEq/L
HBO2 70%
Base Excess −3 mmol/L
Lactate 2.1 mmol/L

*K/μL,* kilogram per microliter; *g/dL*, grams per deciliter; *mmol/L*, millimoles per liter; *mg/dL*, milligrams per deciliter; *u/L,* units per liter; *HPF*, high power field; *mmHg*, millimeters of mercury; *mEq/L*, milliequivalents per liter.

**Table 2:** Approach to altered mental status.

Initial Assessment
Hypoxia
Hypercapnea
Hypotension/Shock
Hypoglycemia
Hypothermia
Hyperthermia
Secondary assessment
Primary neurologic
Intracranial hemorrhage
Stroke
Seizure
Toxic/Metabolic
Alcohols (ethanol & toxic alcohols)
Recreational drugs
Medication toxicities
Carbon monoxide
Hypo- or hyperglycemia
Hypo- or hyperthyroidism
Electrolytes (sodium, potassium calcium, etc.)
Acidemia
Uremia
Infectious
Meningitis
Encephalitis
Pneumonia
Urinary tract infection
Bacteremia
Sepsis
Other
Psychiatric
